# A lifestyle intervention during pregnancy to reduce obesity in early childhood: the study protocol of ADEBAR – a randomized controlled trial

**DOI:** 10.1186/s13102-020-00198-5

**Published:** 2020-09-10

**Authors:** Nina Ferrari, Laura Schmitz, Nikola Schmidt, Esther Mahabir, Patricia Van de Vondel, Waltraut M. Merz, Walter Lehmacher, Stephanie Stock, Konrad Brockmeier, Regina Ensenauer, Tanja Fehm, Christine Joisten

**Affiliations:** 1 Cologne Centre for Prevention in Childhood and Youth/ Heart Centre Cologne, University Hospital of Cologne, Kerpener Str. 62, 50937 Cologne, Germany; 2 Department for physical activity in public health, Institute of Movement and Neurosciences, German Sport University Cologne, Am Sportpark Müngersdorf 6, 50933 Cologne, Germany; 3 Comparative Medicine, Center for Molecular Medicine, University of Cologne, Robert-Koch-Str. 21, 50931 Cologne, Germany; 4 Cologne Center for Musculoskeletal Biomechanics, Medical Faculty, University of Cologne, Joseph-Stelzmann-Str. 9, 50931 Cologne, Germany; 5 Hospital Porz, Urbacher Weg 19, 51149 Cologne, Germany; 6 Department of Obstetrics and Prenatal Medicine, University Bonn Medical School, Sigmund-Freud-Str. 25, 53105 Bonn, Germany; 7 Department of Biometry (IMSIE), Faculty of medicine, University of Cologne, Kerpener Str. 62, 50937 Cologne, Germany; 8 Cologne Institute for Health Economics and Clinical Epidemiology, The University Hospital of Cologne, Gleueler Strasse 176 - 178/II, 50935 Cologne, Germany; 9 Department of Paediatric Cardiology, Heart Centre Cologne, University Hospital of Cologne, Kerpener Str. 62, 50937 Cologne, Germany; 10 Department of General Pediatrics, Neonatology and Pediatric Cardiology, University Children’s Hospital, University of Düsseldorf, Moorenstr. 5, 40225 Düsseldorf, Germany; 11 Institute of Child Nutrition, Max Rubner-Institut, Haid-und-Neu-Str. 9, 76131 Karlsruhe, Germany; 12 Department of Gynecology and Obstetrics, University Hospital Düsseldorf, University of Düsseldorf, Moorenstr. 5, 40225 Düsseldorf, Germany

**Keywords:** Pregnancy, Intervention, Exercise, Perinatal programming, Maternal, Paternal, Biomarker

## Abstract

**Background:**

The prevalence of obesity in childhood is increasing worldwide and may be affected by genetic factors and the lifestyle (exercise, nutrition behavior) of expectant parents. Lifestyle factors affect adipokines, namely leptin, resistin, and adiponectin as well as cytokines such as tumor necrosis factor alpha (TNF-α) and interleukin-6 (IL-6), which are involved in the regulation of maternal metabolic homeostasis, glucose metabolism, and the development of insulin resistance, metabolic syndrome, gestational diabetes mellitus, and hypertension. However, studies focusing on the effect of exercise or a combination of parental exercise and nutrition on the above-mentioned markers in newborns (venous cord blood) and especially on the long-term development of infants’ weight gain are lacking. The study will investigate the effects of a multimodal intervention (regular exercise, diet) on parental and childhood adipocytokines (leptin, resistin, adiponectin, TNF-α, IL-6, BDNF). The effect of a lifestyle-related change in “fetal environmental conditions” on the long-term weight development of the child up to the age of two will also be assessed.

**Methods/design:**

A randomized multi-center controlled trial will be conducted in Germany, comparing supervised aerobic and resistance training 2x/week (13th to 36th weeks of gestation) and nutritional counseling (6th to 36th weeks of gestation) during pregnancy with usual care. Thirty women (pre-pregnancy Body Mass Index ≥25 kg/m^2^, 6th–10th week of gestation) will be included in each group. Maternal anthropometric and physical measurements as well as blood sampling will occur at the 6th–10th, 13th–14th, 21st-24th, and 36th week of gestation, at delivery as well as 8 weeks and 24 months postpartum. Neonatal measurements and umbilical blood sampling will be performed at birth. Maternal and infants’ weight development will be assessed every 6 months till 24 months postpartum. A difference in childhood BMI of 1 kg/m^2^ at the age of two years between both groups will be assumed. A power size of 80% using a significance level of 0.05 and an effect size of 1.0 is presumed.

**Discussion:**

A better understanding of how lifestyle-related changes in the fetal environment might influence infants’ outcome after two years of life could have a profound impact on the prevention and development of infants’ obesity.

**Trial registration:**

The trial is registered at the German Clinical Trial Register (DRKS00007702); Registered on 10th of August 2016; retrospectively registered https://www.drks.de/drks_web/navigate.do?navigationId=trial.HTML&TRIAL_ID=DRKS00007702

## Background

The prevalence of overweight (Body Mass Index ≥25 kg/m^2^) and obesity (Body Mass Index ≥30 kg/m^2^) is increasing worldwide in every age group [1]. In Germany, the number of obese women of child-bearing age has increased especially over the last 25 years from 8.1% in 1990/1991 (25–34-year-old women) to 17.3% (30–44-year-old women) in 2014/2015 [2–4]. When obese women become pregnant they are at increased risk of several perinatal and postpartum complications such as excessive weight gain, gestational diabetes, preeclampsia, pregnancy-induced hypertension or fetal macrosomia [5, 6]. In addition, maternal overweight, excessive weight gain, and gestational diabetes during pregnancy are associated with obesity in childhood and adolescence in the child [7, 8]. Furthermore, children of overweight mothers are at increased risk for long-term metabolic dysfunctions such as hypertension, insulin resistance or metabolic syndrome [8, 9].

Epidemiological, clinical (animal), and experimental studies suggest that maternal lifestyle during pregnancy influences the metabolic, pro-inflammatory, and neurotropic pattern of the unborn child [10–12]. For instance, lifestyle factors have shown to affect adipokines, namely leptin, resistin and adiponectin as well as cytokines such as tumor necrosis factor alpha (TNF-α) and interleukin 6 (IL-6) [13, 14]. These, in turn, are involved in the regulation of maternal metabolic homeostasis and glucose metabolism, and the development of insulin resistance, metabolic syndrome, gestational diabetes mellitus, and hypertension [13].

Reports show that dietary patterns are associated with adiponectin, leptin, and insulin resistance [14–16]. For example, high intakes of fat are negatively associated with adiponectin levels and positively associated with leptin concentrations during pregnancy [16].

However, there are few reports about the interplay of lifestyle factors, in particular maternal exercise, during pregnancy and their effects on biomarkers in mother and offspring [17, 18].

Moreover, Reilly et al. [19] and Pato et al. [20] documented that paternal overweight is another risk factor for the development of juvenile obesity. In a cohort study among 4871 mothers, fathers, and their children, Gaillard et al. [21] evaluated the associations of paternal as well as maternal pre-pregnancy body mass index (BMI) with childhood body fat distribution and cardiometabolic outcomes. It was found that a higher maternal and paternal pre-pregnancy BMI was associated with an adverse cardiometabolic profile in offspring, although stronger associations were present for maternal pre-pregnancy BMI. These findings were confirmed by Santos Ferreira et al. [22]. In the last decade, research increasingly focused on epigenetic effects of lifestyle factors on offspring. In a systematic review, Dunford and Sangster [23] documented that newborns from obese fathers displayed altered methylation overall and significant hypomethylation at the Insulin-like Growth Factor 2 gene. Furthermore, altered offspring DNA methylation levels were associated with high maternal pre-pregnancy BMI [23]. Altered methylation outcomes at multiple imprint regulatory regions in children born to obese parents, compared with children born to non-obese parents, were also found by Soubry et al. [24]. They conclude that parental lifestyle factors and overnutrition have a preconceptional influence on the (re)programming of imprint marks during gametogenesis and early development [24].

Beside these findings, there is a lack of studies focusing on the effect of exercise or a combination of parental exercise and nutrition on the above-mentioned markers in newborns (venous cord blood) and especially on the long-term development of infants’ weight gain. Most previous studies have investigated maternal lifestyle changes but did not take paternal influences into account [25, 26]. Additionally, most of these interventions only used counseling sessions and information brochures [27, 28], and because of substantial methodological variations (e.g. content, duration, frequency, outcome parameters) results are inconsistent [29]. Paternal or transgenerational effects have predominantly been investigated in animal models with a main focus on nutrition [30–32]. In humans, only birth cohort studies have examined paternal lifestyle factors in combination with selected blood samples [33].

Based on the sparse data available, we designed a multi-center, two-arm, randomized controlled trial to evaluate the effect of a multi-component, family-based program during pregnancy on maternal, paternal, and neonatal biomarkers as well as how lifestyle-related changes in the fetal environment might influence infants’ outcome after two years of life. More specifically, the importance of a healthy lifestyle, in particular regular exercise and diet, will be investigated. The aim of this study is to examine the influence of a lifestyle intervention in overweight/obese mothers and fathers on parental metabolic functions and infants’ weight two years after birth. The purpose of this paper is to present the design and methodology of the ADEBAR (Adiposity prevention through an exercise- and nutrition-based family program) study.

## Methods/design

### Study design and setting

**Fig. 1:**
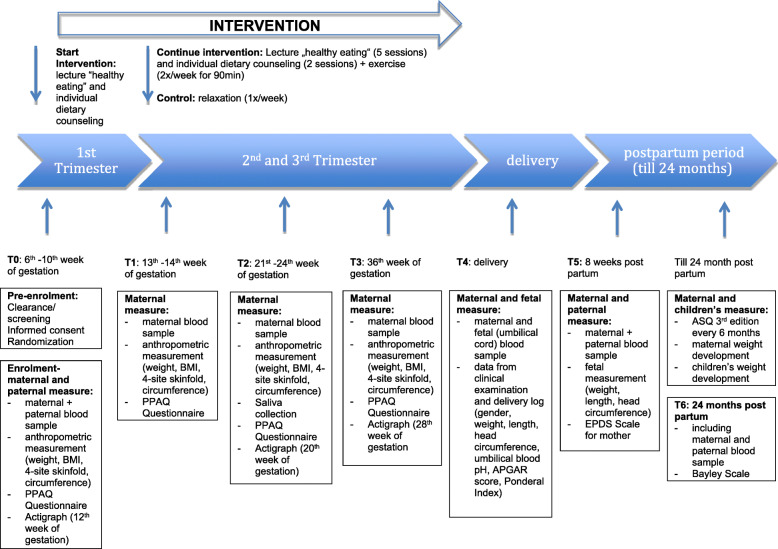
Schematic Timeline of the ADEBAR study

**Fig. 2:**
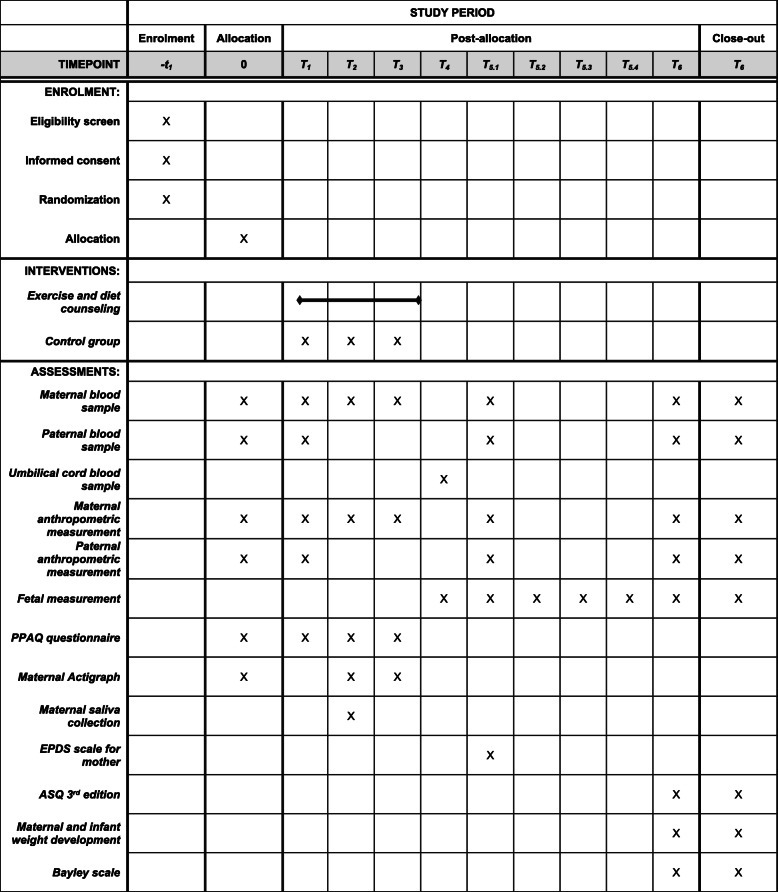
Schedule of enrolment, interventions, and assessments

The ADEBAR study is a multi-centre, two-arm, randomized controlled trial (intervention versus control group) with the primary purpose of prevention. The program will take place at three hospitals in the Rhine region (Cologne, Düsseldorf, and Bonn) in Germany. The intervention period will last approximately 30–32 weeks (8 months) depending on the date of delivery. Mothers, fathers, and their offspring are being followed until the infant is two years of age. A schematic timeline and SPIRIT timetable of the study is presented in Fig. 1 and Fig. 2.

We will perform a block randomization with a block size of six including an equal number of participants for all groups within each block (3:3). This ratio was chosen as it is expected to reduce bias and allocate participants evenly between the intervention and the control group [34].

### Participants

#### Inclusion and exclusion criteria

Women with a pre-pregnancy BMI [35] of ≥25 kg/m^2^, a singleton pregnancy, and a gestational age between 6 and 8 weeks, who were previously sedentary, are without pre-existing conditions such as heart disease, and who are currently not consuming alcohol or using medication for mental health disorders, will be recruited. At 13 weeks of pregnancy, women will receive a physician’s clearance to participate in physical activity. For further participation, they should have no contraindications to exercise during pregnancy according to the American College of Obstetrics and Gynecology [36]. Women with pre-existing diabetes, hypertension, or comorbidities known to affect fetal growth will be excluded from the study as well as women who are not proficient in German. Monthly obstetric check-ups will ensure maternal and fetal well-being.

#### Drop-out criteria

Women with complications during pregnancy as assessed by participating obstetricians and physicians will be excluded from the study. Furthermore, women who will give birth pre-term (delivery before 37th week of gestation), who will not have participated regularly in the program (less then 80% of the units) or who will have missed blood tests (at the beginning of the program or 36th week of gestation) will be excluded.

#### Sample size

Power analysis was done based on recommendations of weight gain in German children at 24 months after birth [37]. Normal weight gain (50th percentile) after subtraction of birth weight is nine kilograms at the age of 24 months. The BMI shows a difference of 1 kg/m^2^ at this time point (50th percentile). Therefore, a difference of 1 kg/m^2^ at the age of two years between the intervention and the control group will be assumed. To detect this difference in proportions with 80% power (GPower analysis) using a significance level of 0.05 and an effect size of 1.0, 48 women are needed. Calculating with a drop-out rate of 25% during the program, 60 women are required and planned to be recruited for this study.

#### Recruitment

Participants will be recruited by hospital- and community-based obstetricians in Cologne, Düsseldorf, and Bonn. To inform eligible women about the study, an information brochure with inclusion criteria and overall information will be used by hospital staff and obstetricians. If a woman shows interest, the recruiting centre will inform the project coordinator to contact her and provide further information about the randomization (intervention or control group). The date of the first enrolment was the 21st of June 2016. The recruitment is ongoing.

### Intervention

Depending on the place of recruitment, the nutrition counseling and exercise program will take place either in Cologne, Bonn or Düsseldorf in a conference room or at a gym, respectively. The intended group size will be 30 participants.

#### Nutrition counseling

Every nutrition counseling will be an extra appointment and is not part of the usual prenatal care appointments.

Pregnant women and their partners will be invited to a lecture about healthy diet during pregnancy at 8 weeks of gestation. This lecture will be followed-up by a 24 h dietary recall [38] to analyze individual energy intakes. This data will be entered and processed using the Prodi-Software® 6.2 (Science publishing society Stuttgart 2013, Germany) to calculate daily amounts of foods and nutrients. Based on this data, individual dietary counseling for the mother and, if possible, for the father will take place at 10 to 12 weeks of gestation. Individual counseling will be repeated at 16 and 24 weeks of gestation based on further 24 h dietary recalls. Additionally, group dietary counseling will consist of five sessions (weeks 14, 20, 30 and 36 of gestation, 8 weeks postpartum) and will be based on recommendations from the Network “Healthy Start – Young Family Network” [39]. Each session will last 60 min and will be led by a specially trained dietician.

After successful participation in the intervention, the families will receive a shopping voucher.

#### Exercise protocol

The exercise program is not part of the usual prenatal care appointments. From the 13th to at least the 36th week of gestation, women will carry out a supervised exercise program (aerobic exercise and strength training) twice a week for 90 min. Resting blood pressure will be measured in the first 10 min of each session before starting the exercise protocol. Every exercise session will include a 10–15 min gradual warm-up and a 10–15 min cool-down including low intensity calisthenics, relaxation and stretching (avoiding muscle pain) of most muscle groups (upper and lower limbs, shoulder and trunk) as well as 10 min of pelvic floor exercises. The main part of the session will last at least 40 min. Depending on the women’s condition, the main part will be devided into two to three parts of 20 or 15 min, respectively, with a little break in between the parts to avoid physical overload. The duration of the parts may be gradually increased as the intervention progresses.

Aerobic exercise sessions will consist of Nordic walking and walking. Each woman will wear a portable heart rate monitor (POLAR Electro Oy, Kempele, Finnland) during each session to stay within the recommended heart rate zone reported by Davenport et al. [40]. Furthermore, the talk test will be used to ensure that exercise is performed at moderate intensity [36].

Strength training will consist of exercises targeting major muscles groups in the upper body, trunk, and lower body by using resistance bands and light dumbbells. In total, the participants will perform two to three sets with 12–15 repetitions of 8–10 muscular strength exercises [41]. The workload and exercises will be documented for each woman on an exercise sheet. The intensity of the exercises will be set according to each woman’s perceived effort using the Borg scale [42]. Participants will progressively increase the resistance of each exercise. In addition, women will be encouraged to take up leisure-time activities such as walking for an additional 30 min per week to reach the recommended level of 210 min of exercise per week [36, 43].

### Control group

The intended group size will be 30 participants. Women allocated to the control group will neither be encouraged nor discouraged from exercising. They will undergo routine antenatal care and receive standard nutrition and activity guidelines from their obstetricians. To improve compliance, women from the control group will participate in a relaxation class, which includes breathing techniques and guided meditation once a week. The control group will undergo the same physical assessments as the intervention group.

#### Primary outcome

The primary outcome of this study will be the BMI of the infant at two years after birth assessed by German percentile [44].

#### Secondary outcomes

Secondary outcomes will be assessed during pregnancy and postpartum and will include:

- The course of pregnancy (weight gain according to the recommendations of the Institute of Medicine (IOM) [45], manifestation of pregnancy-induced diseases, mode of delivery)
- Anthropometric data of the mother and the father (weight, height, BMI, circumferences, body fat distribution)
- Anthropometric data and health status of the new-born (birthweight, length, head circumference, umbilical artery pH value, Apgar scores at one, five and ten minutes)
- Cord blood biomarkers in the newborn (insulin, leptin, adiponectin, resistin, BDNF, TNF-α, IL-6, CRP, fetuin A)
- Maternal and paternal biomarkers (insulin, leptin, adiponectin, resistin, BDNF, TNF-α, IL-6, CRP, fetuin A) during and after pregnancy
- Maternal cortisol level in the saliva at mid-pregnancy
- Postnatal depression (using the Edinburgh Depression Scale) 8 weeks after birth
- Infant development (age-appropriate development of fine and gross motor skills, communication, personal and social skills)

### Outcome measures

#### Maternal measurements

Maternal anthropometric measurements of weight, height, BMI, skinfolds, and mid-arm and mid-thigh circumference will be assessed prior to the start of the program (week 8 of gestation), at weeks 14, 24, and 36 of gestation, at 8 weeks postpartum, and at 12 and 24 months postpartum.

Maternal body weight will be measured to the nearest kg using a digital scale (Tanita Corp., Tokyo, Japan) with the participant wearing casual clothing and no shoes. Maternal weight gain will be defined as the difference between self-reported pre-pregnancy weight and the last weight recorded before delivery. The IOM recommendations [45] will be used to classify excessive weight gain. Height will be measured to the nearest 0.1 cm using a metal stadiometer. Mid-arm and mid-thigh circumference will be measured to the nearest 0.1 cm with a non-extensible, flexible tape on the right side of the body. Skinfold thickness will be assessed by an observer using a Harpenden skinfold caliper (John Bull British Indicators Ltd., Harpenden UK) with a constant pressure of 10 g/mm^2^. The procedure will be carefully standardized and each measurement will be made in triplicate on the right side of the body. Four points will be measured to the nearest 0.2 mm at the triceps, thigh, subscapular and suprailiac site; the results will be averaged and incorporated into the eq. [46] to estimate percent body fat [47].

#### Paternal measurements

Paternal anthropometric measurements of weight, height, BMI, skinfolds, and circumferences will be assessed prior to the start of the program (week 8 of gestation), after delivery at 8 weeks postpartum, as well as two years after delivery following the same procedures as described above.

#### Blood sample

Maternal serum profiles will be assessed at eight time points after an overnight fast (> 10 h): before (week 8 of gestation), during (weeks 14, 24, 36 week of gestation), and after the intervention (labor, and at 8, 12 and 24 weeks postpartum). Paternal serum profiles will be assessed at three time points after an overnight fast (> 10 h): before (week 8 of gestation) and after intervention (at 8 weeks and 24 months postpartum). Umbilical cord blood samples will be collected from the placental part of the umbilical cord immediately after clamping.

Maternal, paternal, and umbilical cord blood samples will each be collected into separate 7.5 ml Sarstedt® gel vacutainers. The samples will be centrifuged within the next four hours at 2500 x g for 10 min at 4 °C. After centrifugation, serum will be separated into 1.6 ml non-pyrogenic, non-mutagenic cryoPure aliquots and stored at − 80 °C until analysis. All samples will run in duplicates and in case of a coefficient of variation of > 5%, a third measurement will be performed.

Serum concentrations of glucose, total cholesterol, triglycerides, HDL-cholesterol, and LDL-cholesterol will be analyzed by colorimetry with a Cobas Integra analyzer (Roche Diagnostics, Germany) according to the manufacturer’s instructions. Insulin will be measured by radioimmunoassay (Roche Diagnostics Germany). Serum irisin and serum fetuin-A will be detected using a human ELISA kit (Phoenix Pharmaceutical, Burlingame, CA/USA; Epitope Diagnostics, San Diego, CA/USA). Adiponectin, CRP, leptin, resistin, BDNF and TNF-α will be investigated by a multiplex immunoassay (2Plex and 4Plex) from eBioscience according to the manufacturer’s instructions. Serum IL-6 will be analyzed using single high-sensitive human ELISA kit (eBioscience).

#### Saliva collection

Participants will be provided with a package containing written instructions for saliva collection and Salivette collection devices (Sarstedt, Nümbrecht, Germany). Three saliva samples will be collected on one morning: one immediately upon awakening without rinsing the mouth with water, one 15 min and one 30 min after. To avoid contamination of saliva, participants will be asked not to brush their teeth, drink or eat at least 60 min before sampling.

Saliva samples will be centrifuged at 4 °C (1,000 rpm, 2 min; Hettich MR22) and stored at − 80 °C until assayed. The samples will be assayed for salivary-free cortisol as a duplicate in a single assay batch per participant via commercially available enzyme immunoassay (IBL International GmbH).

#### Measurements at birth

Within two hours after delivery, midwives will measure birth weight to the nearest 10 g using an electronic scale and measure recumbent crown-heel length to the nearest 0.5 cm. Additionally, head circumference, Apgar scores at 1, 5, and 10 min, as well as umbilical blood pH will be obtained from the hospital delivery logs, clinical examination, and medical records. To assess neonatal leanness, the neonatal Ponderal index will be calculated as PI = 100x(kg/m^3^). Gestational age at birth will be calculated from the last menstrual period and verified by first-trimester ultrasound measurements. Small (SGA) and large for gestational age (LGA) neonates will be defined as live-born infants below the 10th or above the 90th percentile of birthweight for gestational age, respectively, according to German reference curves [48].

#### Physical activity assessment

The Actigraph wGT3X-BT (Actigraph, Florida, US) will be used to determine physical activity levels based on total physical activity and step counts at three points in time over 3 days (weeks 12, 20, and 28 of gestation) from all participants (intervention and control group). Activity counts, recorded every minute, will be added up to provide total counts per day and converted to metabolic equivalent of tasks (METs) in specific intensity levels [49]. Furthermore, we will use the Pregnancy Physical Activity Questionnaire (PPAQ) as described by Chasan-Taber et al. [50] for all participants to detect physical activity at the beginning of pregnancy (week 8 of gestation), during pregnancy (week 14, 24 of gestation) and at the end of pregnancy (week 36 of gestation). Self-reported time spent in each activity will be multiplied with its intensity to obtain average weekly energy expenditure (MET-h·wk.^− 1^) from each activity.

#### Follow-up

Infants’ weight, height, and head circumference will be obtained from examination records after 1, 4, 8, 12, and 24 months. The Ages and Stages Questionnaire (ASQ 3rd Edition) will be used to systematically screen the children for developmental and social-emotional delays in the crucial early years of life [51]. Five domains of development, including communication, gross motor, fine motor, problem-solving, and adaptive skills will be assessed through this questionnaire every four months (at 4, 8, 12, 16, 20, and 24 months postpartum). In conjunction with the questionnaire at 24 months, the Bayley Scale of infant and toddler development, 3rd edition (German version), will be used to assess motor (fine and gross) skills, language and cognitive development at this time point.

Furthermore, by using the Edinburgh Postnatal Depression Scale (EPDS Scale), postnatal depression will be recorded at weeks 6–8 after delivery [52]. In addition, questionnaires will be used to analyze maternal and paternal lifestyles after the intervention based on the German Health Interview and Examination Survey for Adults [53].

#### Statistics

The main variable (BMI at 24 months) will be analyzed by covariance analyses evaluated by the factor group, breastfeeding, and smoking as well as covariances of parental age, paternal BMI, socioeconomic status, breastfeeding, and potential pregnancy-induced diseases (e.g. gestational diabetes mellitus, pregnancy-induced hypertension (PIH)). The significance level alpha will be set at < 0.05 for all analyses. All confidence intervals will be estimated at 95%. Missing data will be calculated according to the LVCF approach (Last Value Carried Forward). Analyses will be carried out as per-protocol-analyzes and intention-to-treat analyses (ITT). Spearman and Pearson correlation procedure will be used to assess relationships between maternal data (physical activity in METs, eating habits, weight gain, blood samples) and neonatal data (birth weight, length, head circumference, Apgar scores, cord blood sample) as well as infants’ development. Multiple linear regressions will be performed for neonatal measures to determine correlations with maternal and paternal lifestyle factors. The model will include group (intervention yes/no) as well as maternal and paternal characteristics as possible confounders. Explained variance (r-squared) for the linear regression model will be reported as model fit statistic.

Statistical analysis will be performed using SPSS 24.0 data-analysis software (Statistical Product and Service Solutions 24.0) and data analysis will be blinded.

#### Data monitoring and management

There will be no Data Monitoring Committee, due to the fact that no high risks for participants are expected. According to Cairns et al. [54] Data Monitoring Committees are only required where survival is a primary endpoint of the trial.

All participants will be recorded via a predetermined code. The anonymized final data will remain at the study center at the German Sport University Cologne and stored and analyzed in a computer system. This system is restricted and can only be opened by password by authorized persons. Identification of the participants will only be possible via the predetermined code by the project manager. All principal investigators will be given access to the cleaned and anonymized data set. It is guaranteed that personal data will not be passed on to third parties.

## Discussion

Overweight and obesity in pregnancy is associated with adverse short- and long-term health effects for both mother and infant. Due to an increasing number of overweight and obese German women of child-bearing age, it is crucial to develop new strategies to prevent obesity in pregnancy and therewith in childhood. Recent studies indicate that, in addition to the mother, the father also plays a pivotal role in the development of childhood obesity. Therefore, we included the father in our intervention program and suggest that future studies should take paternal factors into account.

However, besides the innovations and strengths of our study design there is also a limitation. We are aware that blinding is an important aspect of all studies, which contributes to avoiding and preventing conscious and unconscious bias (for example performance bias or detection bias) in the design and conduction of a trial. Due to our design and recruitment all participants will know which treatment (intervention or control) they will receive. Due to the fact that all participants will be recorded via a predetermined code, at least data input as well as data analysis will be blinded.

To conclude, the main aim of our study is to determine whether regular physical activity and nutritional counseling during pregnancy has an impact on infants’ BMI and infants’ development two years after birth. Our findings will help to detect new influential lifestyle factors during pregnancy which may prevent obesity in early life. Furthermore, the results will provide a basis for new recommendations for action. Therefore, no later than five years after the last recruitment, we will try to deliver the first results and recommendations for sharing purposes.

## Data Availability

Not applicable.

## Abbreviations

ASQ: Ages and Stages Questionnaire
BDNF: brain-derived neurotrophic factor
BMI: body mass index
CRP: C reactive protein
ELISA: Enzyme-Linked Immunosorbent Assay
EPDS: Edinburgh Postnatal Depression Scale
GDM: gestational diabetes mellitus
HDL: high density lipid
IL-6: interleukin 6
IOM: Institute of Medicine
ITT: Intention-To-Treat
LDL: low density lipid
LGA: large for gestational age
LVCF: Last Value Carried Forward
MET: metabolic equivalent of task
PI: ponderal index
PIH: pregnancy-induced hypertension
PPAQ: Pregnancy Physical Activity Questionnaire
SGA: small for gestational age
TNF-α: tumor necrosis factor alpha
